# Blockade of Interleukin-6 Receptor with Tocilizumab During the Acute Phase of Childhood Immune-Mediated Epilepsy can Lead to a Favorable Outcome - an Observational Study

**DOI:** 10.2174/011570159X455475251203194508

**Published:** 2026-01-15

**Authors:** Xiaolu Deng, Jielu Tan, Miriam Kessi, Zhanwei Zhang, Fang He, Ciliu Zhang, Fei Yin, Lifen Yang, Jing Peng

**Affiliations:** 1 Department of Pediatrics, Xiangya Hospital, Central South University, Changsha, China;; 2 Department of Pediatrics, Hunan Intellectual and Developmental Disabilities Research Center, Changsha, China;; 3 Clinical Research Center for Children Neurodevelopmental Disabilities of Hunan Province, Xiangya Hospital, Central South University, Changsha, China

**Keywords:** Tocilizumab, immune-mediated epilepsy, interleukin-6, children, efficacy, safety

## Abstract

**Introduction:**

To explore the role of tocilizumab in treating childhood immune-mediated epilepsy and determine the role of interleukin-6 (IL-6) in its pathogenesis.

**Methods:**

We collected and analyzed clinical information of pediatric patients diagnosed with immune-mediated epilepsy and treated with tocilizumab at Xiangya Hospital.

**Results:**

This study included four males with a median age of onset of 4.3 years. They presented with seizures, fever, and neuropsychiatric manifestations. A combination of different anti-seizure medications (ASMs) and first-line immunotherapy could not control seizures. All patients had elevated levels of IL-6 in their serum and/or cerebrospinal fluid at some points. The median time from disease onset to the first dose of tocilizumab was 25 days. Patients 2 and 3 received tocilizumab in the acute phase of the disease, but patients 1 and 4 received this therapy in the subacute phase. At the last follow-up, patients 2 and 3 had no seizures, while patients 1 and 4 still had chronic epilepsy, but with > 50% reduction of seizure frequency. All patients regained normal cognition.

**Discussion:**

IL-6 may play a role in the pathogenesis of immune-mediated epilepsy, and introducing tocilizumab in the acute phase might be effective in preventing the disease from progressing into the chronic phase.

**Conclusion:**

Tocilizumab is effective for the management of immune-mediated epilepsy in children when given early enough.

## 1 INTRODUCTION

Immune-mediated epilepsy is caused by an autoimmune disease or process [1]. It is characterized by one or more features of encephalitis and the sudden onset of refractory seizures [2, 3]. It is diagnosed when a patient presents with status epilepticus, onset of acute or subacute high-frequency seizures with variable semiologies, multifocal seizures on an electroencephalograph (EEG) that are refractory to antiseizure medications (ASMs), and rapid cognitive decline or behavioral changes [1]. In addition, inflammatory cerebrospinal fluid (CSF) findings (elevated protein, oligoclonal bands, pleocytosis, elevated IgG index) and neuroradiology findings showing inflammation in the mesial temporal regions (edema and T2 hyperintensity) can also provide clues for the diagnosis of this condition [1]. The prevalence of immune-mediated epilepsy in children with epilepsy ranges from 9.5% to 9.7% [4-6]. While the pathogenesis of immune-mediated epilepsy remains to be elucidated, the existence of tumors and infections has been demonstrated to play a role in epileptogenesis [2]. This condition is managed with ASMs, immunotherapies, and surgical removal of a tumor (if applicable) [2]. Although there is no clear data about the prevalence of refractory immune-mediated epilepsy in children, it is estimated that 11- 35% of refractory epilepsy cases could be due to an immune etiology [1]. The administration of immunotherapy in the acute stages of disease in adult patients yields a favorable outcome in approximately 62.00%, reducing seizure frequency and enhancing cognitive function [7]. Nevertheless, we lack knowledge about the most effective immunotherapy choices for children.

Tocilizumab is among the immunotherapies that are being used to treat immune-mediated epilepsy in adults. It is a humanized anti-interleukin-6 receptor (IL-6R) monoclonal antibody [8]. It is increasingly reported to be effective in the treatment of arthritis, adult-onset Still’s disease, arteritis, Behcet’s disease, Castleman disease, interstitial lung disease, Coronavirus Disease 2019 (COVID-19) pneumonia, and neuromyelitis optica spectrum disorder (NMOSD) [9-14]. To date, the U.S Food and Drug Administration (FDA) has not approved the use of tocilizumab for the treatment of epilepsy. Nevertheless, a randomized controlled trial has provided evidence that supports its utilization in adult patients diagnosed with refractory status epilepticus [15]. Furthermore, tocilizumab has been documented as a successful therapeutic intervention for managing adult patients who have developed new-onset refractory status epilepticus (NORSE) and have demonstrated resistance to first-line therapies [16]. Despite the absence of a definitive understanding of the underlying mechanisms of NORSE, a prevailing hypothesis suggests the potential involvement of dysregulation in the innate immune system. Indeed, several NORSE patients exhibit elevated levels of pro-inflammatory cytokines (interleukin (IL)-6, IL-8, IL-10, interleukin-1beta (IL-1β), and tumor necrosis factor alpha (TNF-α) in both the CSF and serum [17-19]. However, no study has explored the role of these pro-inflammatory cytokines in the pathogenesis of childhood immune-mediated epilepsy. Although a recent review provided more evidence in favor of using tocilizumab in NORSE/ febrile infection-related epilepsy syndrome (FIRES) [20], there is no report describing its use in childhood immune-mediated epilepsy. Consequently, we aimed to investigate the role of IL-6 in immune-mediated epilepsy and the efficacy of tocilizumab in managing it in children. Understanding the role of IL-6 in immune-mediated epilepsy can open more avenues for exploration of the pathways involved in its pathogenesis. Understanding the efficacy of tocilizumab in treating childhood immune-mediated epilepsy can guide clinicians to make better immunotherapy choices that improve patient outcomes and quality of life.

## 2 METHODS

This retrospective study was approved by the ethical committee of Xiangya Hospital, Central South University, and followed the rules of the Helsinki Declaration.

The ethical approval number 202310892 was obtained on 19th October 2023. Clinical data were collected retrospectively at our hospital. The parents/guardians of the patients provided informed and written consents.

### 2.1 Participants

All included patients were diagnosed with immune-mediated epilepsy before the age of 14 years, from February 2024 to August 2024 at the Department of Pediatrics, Xiangya Hospital. We included patients who met all of the following criteria: (1) patients diagnosed with new onset immune-mediated epilepsy, (2) patients who received first-line immunotherapies (intravenous methylprednisolone (IVMP) and/or intravenous immunoglobulin (IVIG) and/or plasma exchange (PE)) and tocilizumab, and (3) patients with follow-up > 1 year. We excluded: (1) patients diagnosed with immune-mediated epilepsy but who were treated with other nonsteroidal immunotherapies (including rituximab, cyclophosphamide, mycophenolate, azathioprine, bortezomib and so on), (2) patients diagnosed with antibody-positive autoimmune-associated epilepsy (including anti-N-methyl-D-aspartate receptor (anti-NMDAR), anti- glutamic acid decarboxylase (GAD-65), and so on), and (3) patients with chronic epilepsy (duration of > 3 months from disease onset) when using tocilizumab for the first time.

### 2.2 Diagnostic Criteria

Epileptic seizures and syndromes were defined using the classification of the International League Against Epilepsy (ILAE). We diagnosed immune-mediated epilepsy according to clinical, serologic, or imaging evidence as described before [1]. We evaluated cognition using the Diagnostic and Statistical Manual of Mental Disorders, Fifth Edition (DSM-5), as described in our previous studies [21, 22]. We used observations, clinical interviews, and standardized, age-related rating scales to assess adaptive functioning. However, doctors often made the diagnosis using their own judgment instead of using formal, standardized assessments, especially for young patients [23]. The age-related rating scales used were the Gesell Developmental Schedules for patients aged 2-4 years, and the Wechsler Preschool and Primary Scale of Intelligence Fourth Edition (WPPSI-IV) for patients aged 4-6 years, and 6 years old and above. For patients younger than two years old, doctors used their own judgment to decide how severe the developmental delay (DD) was. Patients with deficits in adaptive and intellectual functioning that began during their developmental period were classified into four groups: mild intellectual disability (ID) when IQ values were between 55 and 70, moderate ID when IQ values were between 40 and 55, severe ID when IQ values were between 25 and 40, and profound ID when IQ values were less than 25. Patients with a developmental quotient (DQ) ranging from 65 to 84 were considered to have mild DD; those with a DQ ranging from 45 to 64 were considered to have moderate DD; and those with a DQ of 44 or less were considered to have severe DD.

### 2.3 Treatment Algorithm

Our patients were given tocilizumab (8-12 mg/kg intravenously) as prescribed in children with epilepsy in the previous studies [24-26].

### 2.4 Data Collection

The data collected comprised clinical manifestations, prodromal symptoms, seizure semiology, seizure severity, EEG results, magnetic resonance imaging (MRI) results, genetic and metabolic tests results, infection and cancer surveillance tests results, CSF and serum analysis results, therapies (ASMs and immunotherapies) used, and dosages. We kept track of the side effects of treatment, epilepsy prognosis, EEG and brain MRI results, and cognitive tests.

### 2.5 Statistical Analysis

Statistical analysis was done using SPSS Version 27 (IBM, Armonk, NY). Categorical data were summarized in the form of frequencies and proportions.

## 3 RESULTS

### 3.1 Demographic and Basic Clinical Information of the Patients

**Fig. (3):**
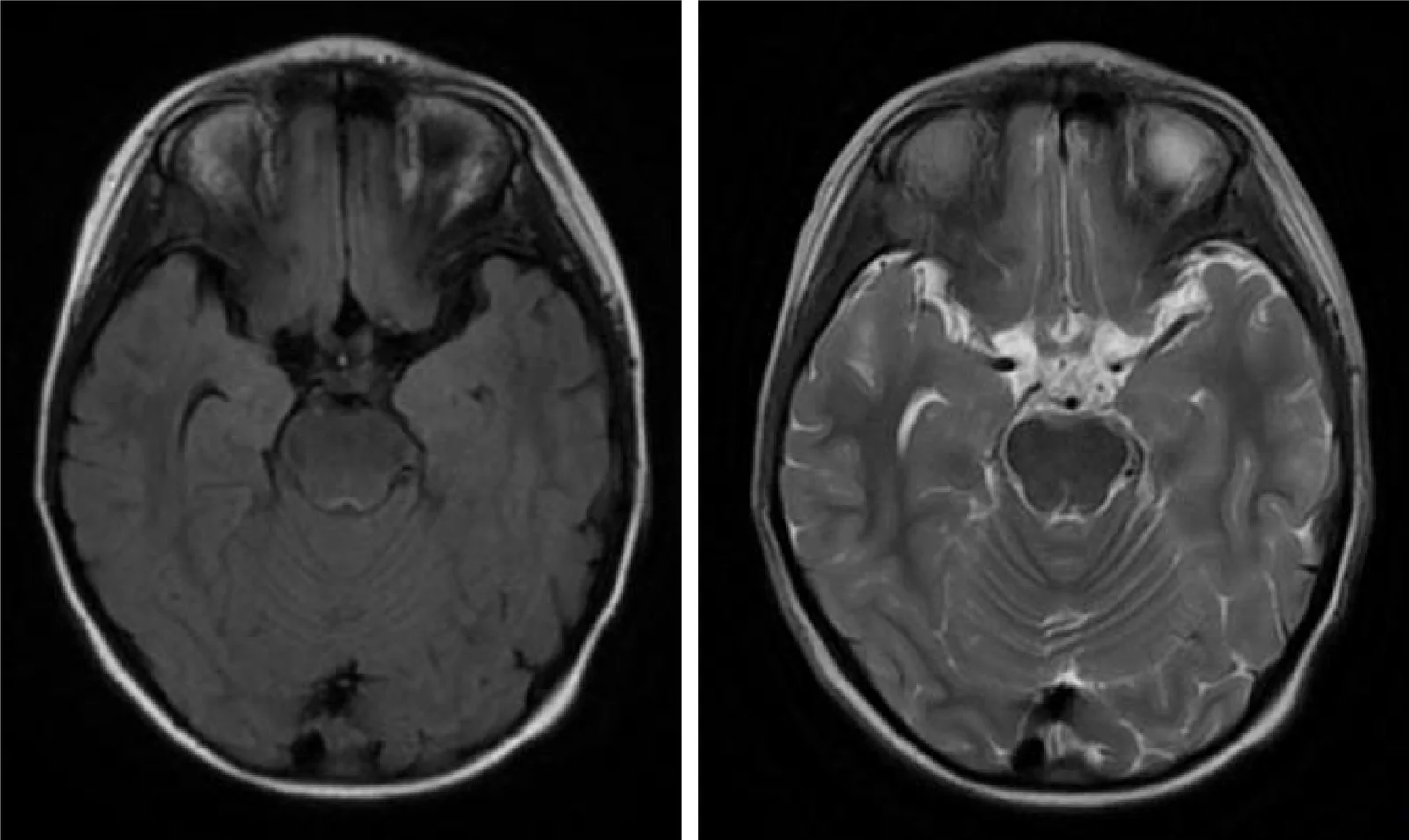
Brain magnetic resonance imaging for patient 4 in the acute phase. There is an increased intensity and edema in the left hippocampus and temporal lobe (T2 and T2 FLAIR).

We recruited four patients diagnosed with immune-mediated epilepsy, all of whom were males. The median age at seizure onset was 4.3 years (range, 1.92-9.16 years). All four patients presented with new-onset focal seizures that originated from different regions, and all had normal development before disease onset. Before the use of tocilizumab, the severity of the seizures was as follows: patient 1’s seizures could last for 10 minutes and occurred 15 times within two hours, while patient 2’s seizures could last 10-20 minutes (dozens of times a day) and occurred 12 times within 4 hours according to the EEG monitoring. Patient 3’s first seizure lasted for 90 minutes; however, he previously experienced shorter-duration seizures that could occur seven times within six hours, according to the EEG monitoring before tocilizumab use. All four patients (100%) had abnormal EEGs. Figs. (**1** and **2**) show representative EEG changes for Patients 1 and 2, respectively, just before the use of tocilizumab. Patient 4’s seizures lasted 4-5 minutes and occurred up to six times on the onset day; the frequency decreased to 2-3 times per week after an administration of IVIG plus IVMP (just before tocilizumab use). In addition to experiencing seizures, all four patients presented with fever and neuropsychiatric changes. Patient 2 presented with Guillain-Barré syndrome (GBS) one month before experiencing seizures. Patient 4 had an abnormal brain MRI (Fig. **3**). At certain points in the disease course, all patients had elevated levels of IL-6 in their serum and/or CSF. Table **1** summarizes this information. Moreover, some patients had abnormal serum levels of IgG (patient 2), IgE (patient 3), and IgM (patients 1, 2, and 3) but normal IgA levels before the use of tocilizumab, as shown in Supplementary Table **1**.

### 3.2 Treatment Strategies and Outcome

On average, all cases received 3.50 ASMs plus a multi-dose regimen of IVIG and/or IVMP before the use of tocilizumab, but these therapies were not effective enough, and seizures progressed rapidly. The median duration from disease onset to the first dose of IVIG was 1.5 (range, 1-4) days. The median duration from disease onset to the first IVMP dosage was 6.5 (range, 2-11) days. The median duration from disease onset to the first tocilizumab dosage was 25 (range, 1-35) days. Within the first half-year, an average of 3.5 tocilizumab cycles was used, which were effective for all cases. Among them, patient 1 became seizure-free after the second cycle of tocilizumab, and his EEG became normal after the fifth cycle. We then tapered off his topiramate and ceased tocilizumab, but he started having seizures again 2 months later, and entered a chronic phase of refractory epilepsy, reminiscent of FIRES. We adjusted dosages of several other ASMs and even tried a ketogenic diet, but his seizures were not controlled well. Then, we tried an additional 5 cycles of tocilizumab together with several repeated IVIG cycles, but still his seizures were not controlled well. In the 14^th^ month of seizure onset, we gave him anakinra. His seizures improved to some degree, but he could still experience 5-10 focal seizures per month at the end of follow-up. Patients 2 and 3 had no seizures again at the end of follow-up, but the former got a respiratory infection once during the follow-up. Although patient 4 improved partly after the first 3 cycles of tocilizumab, he progressed into the chronic phase. He continued to experience a constant frequency of 1 to 2 seizures per month. He was given another 2 cycles of tocilizumab, but there was no significant improvement, so tocilizumab treatment was stopped. At the final follow-up, two cases (50%) had no seizures, one patient who had transient seizure control entered into a chronic refractory phase, and the other had reduced seizure frequency (>50%). Fig. (**4**) shows the EEG of patient 1 when he achieved transient remission (after 5 cycles of tocilizumab). Fig. (**5**) shows the EEG of patient 2 after disease remission. At the last follow-up, four cases (100%) had normal cognition. Table **2** summarizes this information.

## 4 DISCUSSION

This study included four males who were diagnosed with childhood immune-mediated epilepsy and treated with tocilizumab in the acute or subacute phase. The median age at seizure onset was 4.3 years, and all presented with focal seizures originating from multiple regions. Focal seizures occurred after fever and were accompanied by neuropsychiatric changes. An average of 3.5 ASMs were used before the use of tocilizumab, but they could not control the seizures. All four patients had elevated IL-6 levels at some point in the disease. Three patients received different dosages of IVIG and/or IVMP before tocilizumab use, which could not control the seizures. One patient received IVIG, IVMP, and tocilizumab almost at the same time because his seizures were severe and progressed rapidly. The median duration from disease onset to the first dose of IVIG was 1.5 days, and the median duration from disease onset to the first dose of IVMP was 6.5 days. The median duration from disease onset to the first tocilizumab dosage was 25 days. An average of 3.5 cycles of tocilizumab were used in the first half-year of follow-up, and it was effective in all cases. At the last follow-up, two patients were seizure-free, while two still had epilepsy. All patients regained normal cognition. Different timing of tocilizumab administration might lead to different results; an earlier initiation of tocilizumab seems to be associated with a better outcome.

Similar to previous studies [2, 3], our patients presented with new-onset refractory seizures accompanied by viral prodromal symptoms, psychiatric symptoms, subacute progressive cognitive decline, inflammatory signs in the CSF, and brain MRI changes observed in autoimmune encephalitis. Our patients’ EEG findings improved after the administration of tocilizumab, similar to a previous report on NORSE [27]. In addition, it has been shown that tocilizumab can lead to favorable outcomes and Fluorodeoxyglucose Positron Emission Tomography (FDG-PET) changes in FIRES patients [28]. Although none of our cases had a tumor during follow-up, they had an infection that could have triggered the condition, which is consistent with the results of other studies [2]. Notably, lacosamide, which has been reported to be effective in other studies [2], could not help our two patients who tried it. All of our patients received first-line immunotherapies: IVIG and/or IVMP at different times and dosages. However, improvement was not significant until we administered the second-line therapy (tocilizumab), which concurs with a NORSE study that involved seven adult patients [16]. Similarly, a prospective pediatric cohort study reported that, of 15 patients diagnosed with immune-mediated epilepsy who were treated with ASMs and dexamethasone oral pulse therapy, 7 (46.66%) relapsed [29]. This suggests that ASMs and first-line immunotherapies alone are not adequate to produce a favorable outcome.

Tocilizumab has been reported to be effective in pediatric and adult patients diagnosed with FIRES/NORSE [30]. In a cohort of 74 patients diagnosed with cryptogenic NORSE (C-NORSE) who failed to respond to first-line immunotherapy, 68 received second-line immunotherapy (rituximab and tocilizumab). Consequently, 83.8% regained consciousness within a median of 30 days, and 50% achieved a good outcome (mRS ≤ 2) at 2 years [31]. An adult patient diagnosed with C-NORSE was refractory to ASMs and first-line immunotherapies, including high-dose corticosteroids and PE, but status epilepticus disappeared after the administration of tocilizumab at 34-41 days [32]. Another adult patient diagnosed with C-NORSE had a favorable outcome after receiving intravenous cyclophosphamide at day 19 of disease onset [33]. Reported effective therapies for the chronic phase of FIRES of some children and adult patients include minocycline, anakinra, cannabidiol, and a ketogenic diet [34]. An early concurrent administration of intrathecal dexamethasone and anakinra/tocilizumab in two children with FIRES allowed them to maintain freedom from seizures [35]. One study involving 58 pediatric patients diagnosed with FIRES compared a tocilizumab group (n=23) with a control group (n=35) and showed that the use of tocilizumab was associated with higher rates of recovery of consciousness, as well as favorable Pediatric Cerebral Performance Category scores [24]. This study also showed that the group that responded to tocilizumab treatment had an earlier administration of tocilizumab, less time on mechanical ventilation, less time in the intensive care unit, and higher consciousness recovery rates [24]; all findings that concur with recommendations in a review [36]. Anakinra and tocilizumab have both been recognized as effective treatments in the chronic phase of FIRES in some children [37].

Our study shows that tocilizumab might be related to different outcomes depending on the timing of its administration: in the acute or subacute phase. Patients 2 and 3 received tocilizumab in the acute phase of the disease, while patients 1 and 4 received it in the subacute phase of the disease (they came to our hospital after one month of disease onset). Patients 1, 2, and 4 received tocilizumab after first-line immunotherapies failed, while patient 3 received it simultaneously with first-line immunotherapies. Seizures were controlled rapidly after the first cycle of tocilizumab for patients 1, 2, and 3, which concurs with the findings from a study that included 3 FIRES pediatric patients [38]. It is worth noting that patient 1 relapsed and entered the chronic phase (presenting with FIRES-like manifestations) later after attaining seizure freedom for more than 5 months. He had severe and very frequent seizures in a previous hospital and was treated with several first-line immunotherapies as listed in Table **2**. His seizures improved a bit, but they were still very frequent when he came to our hospital. His seizures progressed again while he was at our hospital, and we gave him tocilizumab, which led to dramatic improvement. Patient 2 experienced frequent seizures during the acute phase of disease, but was relieved rapidly with tocilizumab. In the acute and subacute phases of patients 2 and 1, respectively, the frequent severe seizures were probably relieved by the effect of tocilizumab. It is difficult to determine the exact role of tocilizumab treatment in patient 3 because it was administered almost simultaneously with first-line immunotherapies. Similar to patient 1, patient 4 received his first cycle of tocilizumab at the subacute phase of disease, after the partial failure of the first-line immunotherapies before coming to our hospital. However, his situation did not show significant improvement after the first cycle of tocilizumab, similar to a previous report of a child diagnosed with FIRES who received tocilizumab after 36 days of disease [39]. Patient 4’s illness in the acute phase was characterized by even less frequent seizures compared to patients 2 and 3, but he eventually entered the chronic refractory phase, with abnormalities in his brain MRI. His glucocorticoid dose in the acute phase was non-pulse, while the rest of the patients (patients 1,2, and 3) received pulse steroid therapy, which might partially explain the failure to prevent chronic epileptogenesis. The delayed administration of tocilizumab might also partially explain his poor prognosis.

At the end of follow-up, patients 1 and 4 still had chronic refractory epilepsy, which might be because of delayed administration of tocilizumab, as noted in a previous report of a FIRES pediatric patient [39]. Meanwhile, patients 2 and 3 had better outcomes, which might be due to early administration of tocilizumab, similar to another pediatric FIRES study [38]. It has been reported that early administration of intravenous cyclophosphamide and/or tocilizumab, accompanied by ASMs, can result in a better one-year functional status in C-NORSE patients [40]. A recent report showed that tocilizumab is an effective treatment strategy for NORSE even if administered late (several weeks after disease onset) in the course of disease [41]. Another report showed that an adult patient diagnosed with C-NORSE 100 days after admission to the hospital, who was given tocilizumab, experienced a cessation of long-lasting status epilepticus; however, the outcome was poor [19]. In fact, second-line immunotherapy, including anakinra and tocilizumab, is associated with lower mortality and more improvement in Clinical Assessment Scale for Autoimmune Encephalitis (CASE) scores in C-NORSE patients than first-line immunotherapy alone [31]. Likewise, early administration of immunotherapies in autoimmune-associated seizure disorders (anti-LGI1, -NMDAR, and -GAD antibody encephalitis) can help control seizures [42-44]. Specifically, tocilizumab has been reported to be effective in anti-NMDAR encephalitis [45-48], anti-GAD encephalitis [49-51], anti-LGI1 encephalitis [45, 52], anti-CASPR2 encephalitis [53, 54], and anti-amphiphysin encephalitis [45]. Therefore, an early administration of second-line immunotherapies, especially tocilizumab, in immune-mediated epilepsy, FIRES/NORSE, and autoimmune epilepsies, can result in favorable outcomes.

It appears that tocilizumab can halt inflammation-related pathogenesis in the acute stage of disease and prevent chronic epilepsy to some degree. We speculate that early administration of tocilizumab in patient 2 during the acute phase could have stopped the progression of epileptogenesis to the chronic phase. In addition, administering tocilizumab during the subacute phase of the disease might not be able to completely reverse the already formed epileptogenesis in the acute phase. Our observations are supported by findings from previous human and animal model studies. A recent multicenter study revealed that earlier administration of tocilizumab and/or anakinra is associated with a shorter duration of status epilepticus and a decreased frequency of brain atrophy, suggesting an association between NORSE severity and immune activation [55]. According to one study that had 23 pediatric NORSE patients who received second-line immunotherapy (including rituximab, tocilizumab, and anakinra), poor (mRS ≥4) outcome was observed in 14 (61%) patients, and all had chronic epilepsy, probably because of the late initiation of these therapies (especially tocilizumab) [56]. Notably, tocilizumab, anakinra, baricitinib, and intrathecal dexamethasone can be effective in the acute phase of FIRES [38, 57-60], while tacrolimus can be useful in the chronic phase [39]. One study revealed that late introduction of anakinra is associated with long stays in intensive care [61]. One review (mixed children and adult population) revealed that in a few cases, the initiation of anakinra or tocilizumab several years after status epilepticus onset could reduce seizure frequency for 67% of patients [20]. The treatment of drug-resistant seizures (subclinical FIRES) with tocilizumab in mouse hippocampal and temporal cortex acute slices resulted in a delayed onset of epileptiform discharges and reduced ASM-resistant epileptiform activity induced by neuroinflammation [62]. Moreover, tocilizumab exhibited anti-absence, anti-epileptogenic, and antidepressant-like effects in the WAG/Rij rat model of absence epilepsy [63]. Taken together, our study and previous reports in both humans and animal models support the use of tocilizumab in immune-mediated epilepsy and FIRES, especially in the acute phase of the disease, to obtain better outcomes.

We observed that the recommended first-line therapies (IVIG and IVMP) helped our patients to some degree, but were not adequate. Early addition of a second-line immunotherapy (tocilizumab) helped them achieve a better outcome. Tocilizumab blocks the signal transduction of IL-6 to inflammatory mediators, which activate B and T cells [64]. There is a connection between elevated levels of IL-6 and refractory seizures [45, 65]. IL-6 stimulates the differentiation of B cells into plasma cells, and stimulates the differentiation of naive CD4 T cells into IL-17-producing T helper cells (Th-17) [66]. The Th-17 cells cause autoimmune tissue damage and activate CD8+ T cells to produce cytotoxic T cells [66]. Furthermore, monocytes and macrophages with toll-like receptors can produce IL-6 [66]. Although there is a small amount of IL-6 in the central nervous system, stimulation of astrocytes and microglia can increase the production of IL-6 [67, 68]. Notably, increased levels of other cytokines, including IL-Iβ, IL-17, and TNF-α, upregulate IL-6, which decreases hippocampal neurogenesis, increases gliosis, and creates an environment conducive to epileptogenesis [67]. IL-6 is involved in neuroinflammation and may intensify seizure activity and status epilepticus [45, 65, 69, 70]. Therefore, cytokine modulation may alter this neuroinflammation and seizure propagation. Consistent with this, our four patients exhibited elevated levels of IL-6 and demonstrated a somewhat positive response to tocilizumab. In addition, elevated levels of IL-6 in both the CSF and serum have been detected in FIRES/NORSE patients [17-19, 71, 72], similar to our findings. Notably, tocilizumab can decrease CSF IL-6 levels [32, 72].

One of our patients experienced mild side effects from tocilizumab, suggesting that its usage for a short time is safe and effective for immune-mediated epilepsy. Therefore, we recommend using a combination of sufficient and timely first- and second-line immunotherapies, as discussed in the review [2]. However, further research is needed to determine when and on whom to start second-line immunotherapy. Unfortunately, all of our patients were males, which can introduce a potential bias. However, tocilizumab has been reported to effectively treat epilepsy in both adult males and females [15, 28, 35, 41]. The fact that only males were included in our study can be attributed to the small sample size or the nature of the disease [73, 74], just as anti-NMDAR encephalitis is more prevalent in females [75].

## CONCLUSION

Elevated levels of IL-6 in CSF can play a role in the pathogenesis of immune-mediated epilepsy in children. Early administration of tocilizumab in children, especially in the acute phase of this type of epilepsy, can prevent the occurrence of chronic epilepsy. We recommend the initiation of tocilizumab concurrently with ASMs or administering tocilizumab when ASMs fail to control seizures. Our study provides practical insights into early immunotherapy strategies that may improve neurological outcomes in severe immune-mediated epilepsy.

Notwithstanding the robust nature of the present study, it is important to acknowledge its inherent limitations. The study involved a small sample size and male gender alone; however, due to the rarity of immune-mediated epilepsy in children and the new use of tocilizumab, the study design and small sample size were inevitable. Since tocilizumab was administered after or with first-line immunotherapies, it might be difficult to exclude their effects in the treatment response, since some of them can work for a long duration. We could not do a correlation analysis in this study because of our small sample size. Unfortunately, we did not measure the level of tocilizumab in the blood for this cohort. We could not take measurements to control for confounders or bias because each of our patients was treated individually; thus, there is no uniformity of prior treatments or timing of tocilizumab initiation. The follow-up duration was not long enough to observe the efficacy as well as the long-term side effects of tocilizumab. We plan for an extended follow-up of these patients. Meanwhile, prospective multicenter studies and clinical trials are needed to further guide the treatment of immune-mediated epilepsy and to address the limitations observed in our study.

## Figures and Tables

**Fig. (1):**
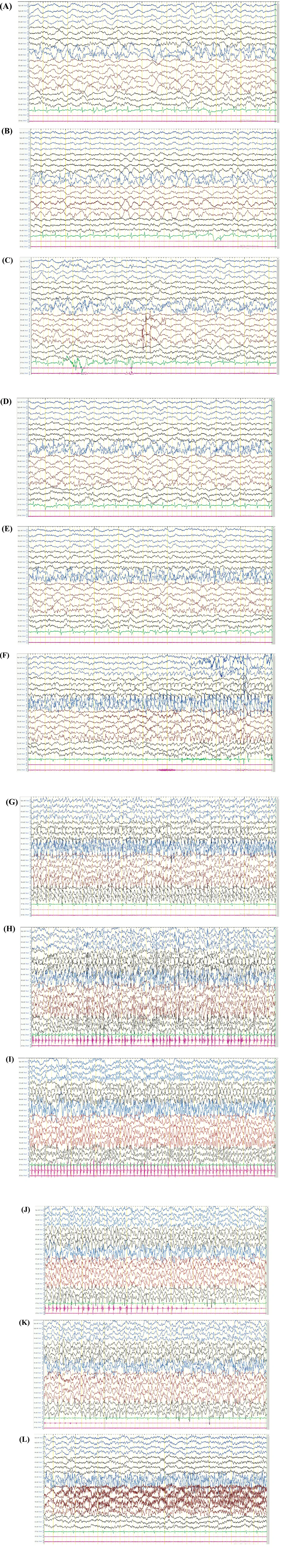
Electroencephalographs of patient 1 in the acute phase. (**A**). The dominant rhythm of the occipital region disappeared and was replaced by diffused slow wave complexes of medium amplitude. Large quantities of sharp and spike-slow wave complexes were continuously emitted in the occipital regions. (**B**). Diffuse slow waves with medium amplitude were continuously observed in the temporal regions, especially on the right side. (**C-L**). Seizure: spikes and spike-slow wave complexes with low-medium amplitude were noticed in the bilateral occipital regions (**C**) → gradually spread to the right middle and posterior temporal regions (**D-E**) → spread to the anterior head and affected the right hemisphere, evolving into a sharp-spike wave/sharp-spike slow wave rhythms (**F**) → affected the midline region (**G**) → seizures spread to the left hemisphere but still dominated the right hemisphere (**H-L**). Compared with the continuous and diffuse abnormal discharges during the interictal period of the electroencephalogram, the beginning and end of the seizure episode are not as clear and obvious.

**Fig. (2):**
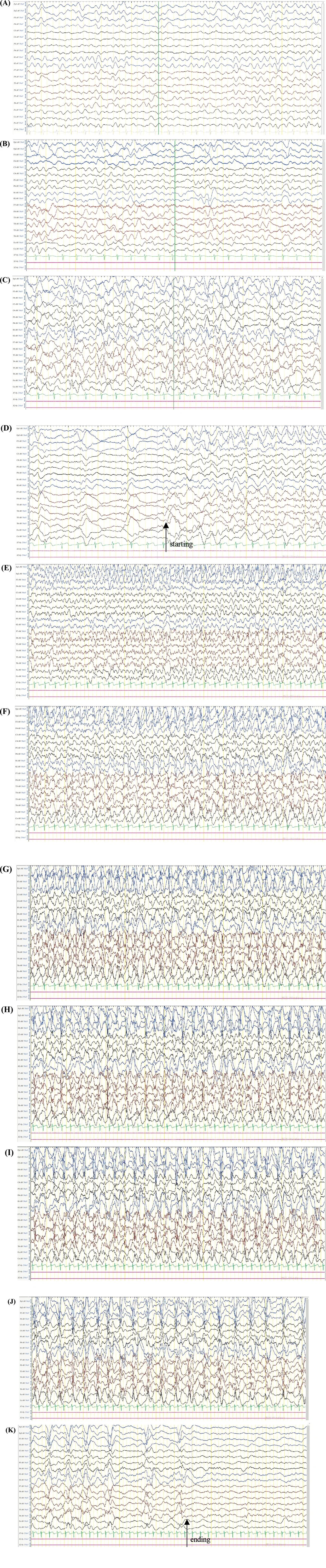
Electroencephalographs of patient 2 in the acute phase. (**A**). Background with the 4-5Hz θ rhythm in the occipital regions. (**B, C**). Numerous multifocal sharp slow wave complexes of medium-high amplitude that occurred in paroxysms or continuously during the waking and sleeping periods. Sometimes slow wave rhythms became paroxysmal in the right temporal regions. (**D-K**). Seizure: the diffused δ waves of medium-high amplitude increased and simultaneously interspersed with sharp α-wave activities in the bilateral frontal/temporal regions (**D**) → the frequency gradually increased while the amplitude decreased gradually, evolving into continuous sharp wave rhythms mainly in the bilateral frontal/temporal regions (especially on the right side) (**E**) → spread to bilateral hemisphere with extremely diffused high amplitude spike-slow wave rhythms (**F-K**).

**Fig. (4):**
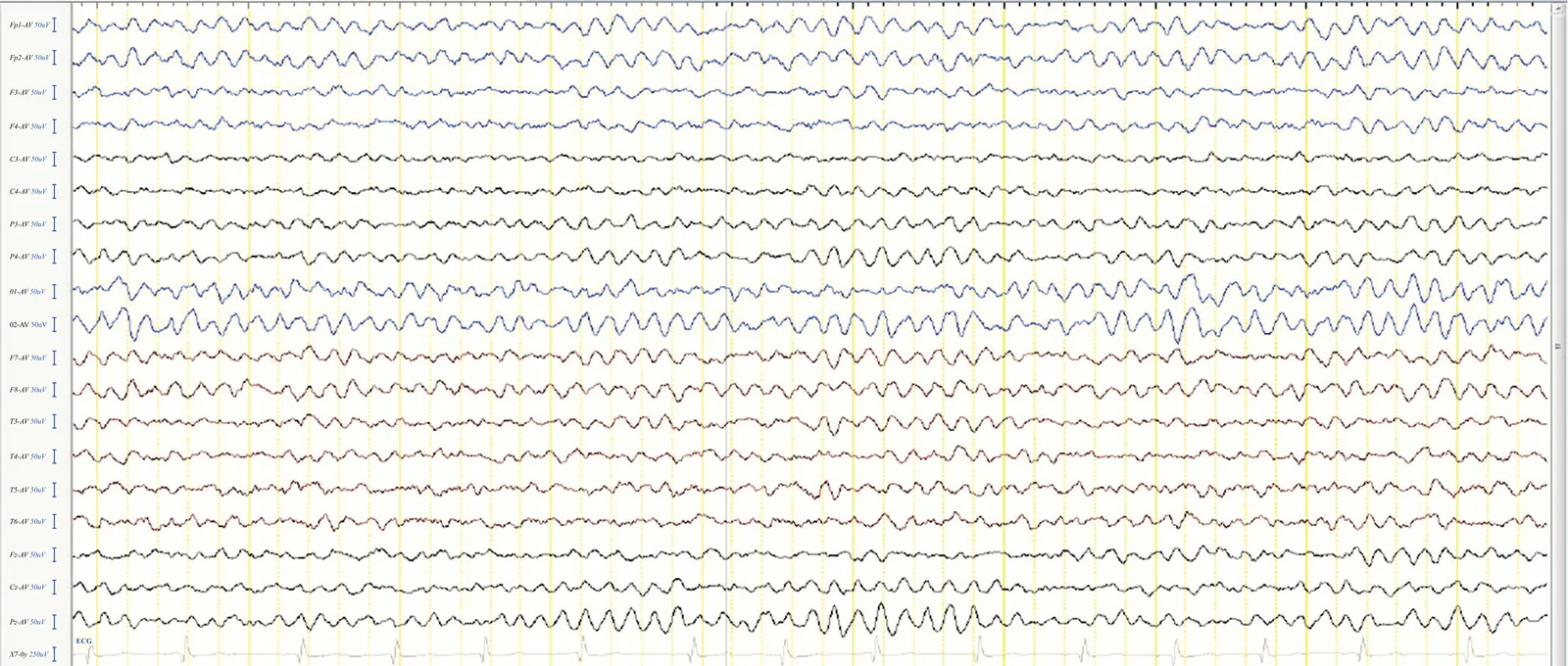
Electroencephalograph of patient 1 after remission. Background after disease remission: 7-8Hz mixed rhythm of θ and α waves in occipital regions.

**Fig. (5):**
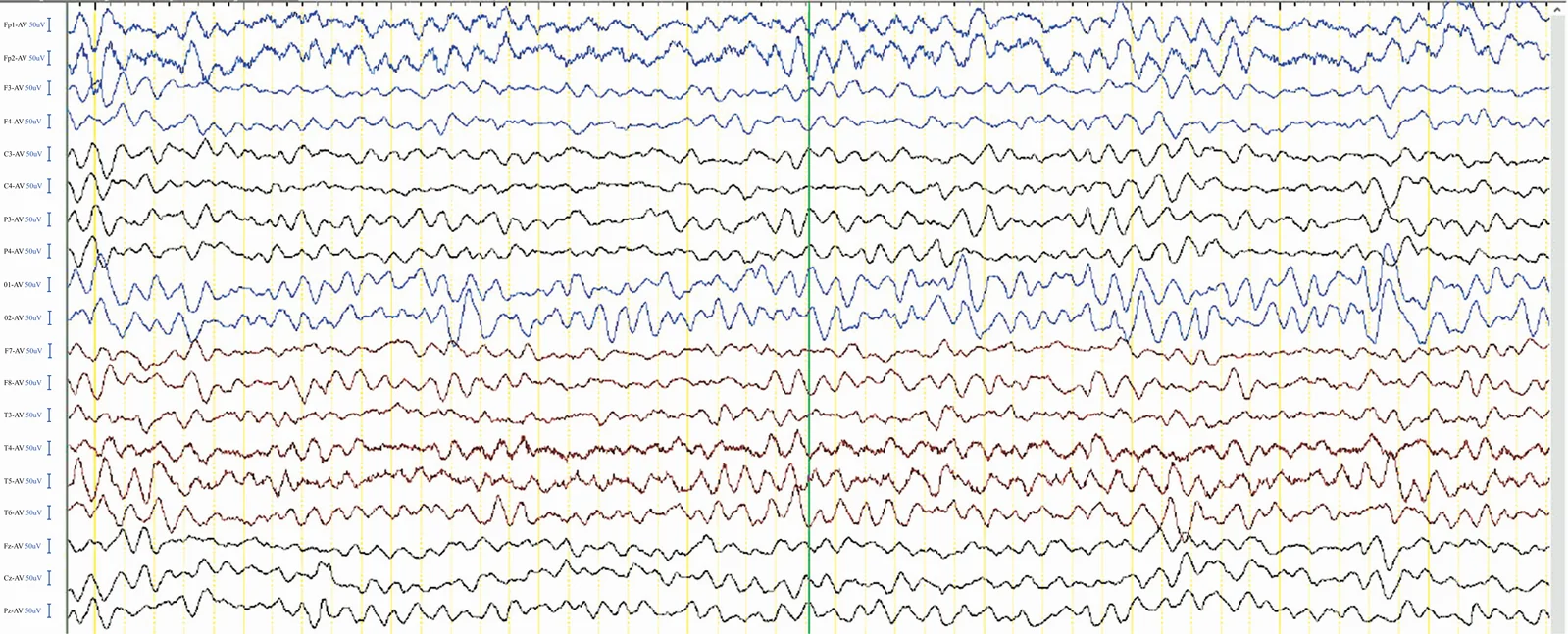
Electroencephalograph of patient 2 after remission. Background after disease remission: 5-7Hz θ rhythms in occipital regions.

**Table 1:** Overview of patient demographics, initial presentations, and baseline clinical findings.

**Clinical Feature**	**Patient 1**	**Patient 2**	**Patient 3**	**Patient 4**
Age of onset	9 years and 2 months	1 year and 11 months	3 years and 11 months	4 years and 8 months
Sex	M	M	M	M
Clinical manifestations	Fever, seizures, agitation, altered mental status, impaired memory, reduced speech, and hyperactivity.	Fever, seizures, aggressiveness, irritability, and hyperactivity.	Fever, headache, vomiting, seizures, and reduced speech	Fever, cough, ear pain, agitation, reduced speech, and unstable gait.
Preceding manifestations	None	He presented with weakness and was diagnosed with Guillain-Barré syndrome around one month before the seizures.	None	None
Seizure semiology	Focal tonic seizures (multifocal).	Focal seizures (multifocal).	Focal seizures (multifocal).	Focal seizures (multifocal).
Disease status in the acute phase	Minimum seizure duration was above 30 minutes, seizures and subclinical seizures happened dozens of times a day according to EEG monitoring.	Seizures could last for 10-20 minutes and could occur dozens of times per day.	The first seizure lasted for 90 minutes. However, he could have seizures 7 times within 6 hours.	Seizures could last for 4-5 minutes and occur 6 times in a day.
Physical examination findings	Could walk slowly, had altered memory, reduced speech, hyperactivity, and shaking of the head and upper extremities.	Unstable gait, aggressive, irritable, and hyperactive	Reduced speech and impaired memory	Agitation, decreased verbalization, and unstable gait.
Investigations	-	-	-	-
EEG (before the first dose of tocilizumab)	Slow style="background-color: slow delta-theta rhythm; sharp slow waves, 15 clinical or subclinical focal seizures during the 2-hour EEG monitoring.	Slow style="background-color: theta rhythm; multifocal sharp and sharp slow waves; 10 focal seizures and 2 subclinical focal seizures during the 4-hour EEG monitoring	Slow background with delta rhythm; large diffused sharp slow waves; 7 clinical or subclinical focal seizures within 6-hour EEG monitoring.	The background was slightly slow, and there were a few diffused slow waves.
MRI in the acute phase	The pineal body was a bit enlarged	Normal	Normal	Edema of the left hippocampus.
Metabolic tests	Normal	Normal	Normal	Normal
Infection surveillance (next-generation sequencing pathogen screening)	Normal	Normal	Normal	Normal
Cancer surveillance	Normal	Normal	Normal	Normal
CSF cell analysis	Elevated protein levels (575.3 mg/L) and WBC (10x10^6^/L), but glucose was normal.	WBC (30x10^6^/L), protein, and glucose were normal	WBC 12x10^6^/L, protein and glucose normal	Normal
CSF and serum analysis (antibodies checked, result)	Antibodies against-NMADR, LGI 1, AMPA1R, AMPA2R, CASPR2, GABA-BR, IgLON5, DPPX, GlyRα1, GABAARα1R, GABAARγ2R, GABAARβ3R, mGLUR5, D2R, MOG, neurexin-3α, GAD65, KLHL11, AK5 (all were negative).	Antibodies against-NMDAR, LGI 1, CASPR2, GABA-BR, AMPA1R, AMPA2R, IgLON5, DPPX, GAD65, mGLUR5, GlyR, D2R, MOG, GFAP, GABAARα1R, GABAARβ3R, NMADR GluN2A, NMADR GluN2B, mGLUR1, mGLUR2, Neurexin-3α, Cava2δ, KLHL11, GluK2, PDE10A, AK5, KCTD16, NCAM1 (all were negative)	Antibodies against-NMDAR, LGI1, CASPR2, GABA-BR, AMPA1R, AMPA2R, IgLON5, DPPX, GAD65, mGLUR5, GlyRα1, D2R, MOG, AQP4, GFAP, CV2 (CRMP5), AGO (all were negative)	Antibodies against-NMDAR, LGI 1, CASPR2, GABA-BR, AMPA1R, AMPA2R, IgLON5, DPPX, GAD65, mGLUR5, GlyR, D2R, MOG, GFAP, GABAARα1R, GABAARβ3R, NMADR GluN2A, NMADR GluN2B, mGLUR1, mGLUR2, Neurexin-3α, Cavα2δ, KLHL11, GluK2, PDE10A, AK5, KCTD16, NCAM1 (all were negative)
Elevated interleukin-6 levels detected during the disease course	21.01 pg/ml (serum)	7.93 pg/ml (serum)	10.86 pg/ml (serum) 15.29 pg/ml (CSF)	23.91 pg/ml (serum) 6.69 pg/ml (CSF)
Genetic tests	Negative	Negative	Negative	Negative

**Abbreviations:** CSF: cerebrospinal fluid, EEG: electroencephalograph, MRI: magnetic resonance imaging.

**Table 2:** Summary of the treatment strategies, prognosis, and outcome.

**Clinical Feature**	**Patient 1**	**Patient 2**	**Patient 3**	**Patient 4**
ASMs used prior to the use of tocilizumab	LEV, TPM, intravenous VPA, continuous IV midazolam, and clobazam (all were ineffective).	LEV, continuous IV midazolam, lacosamide, and clobazam (all were ineffective).	Continuous IV midazolam, intravenous then oral VPA (all were ineffective)	Lacosamide, LEV, continuous IV midazolam (all were ineffective)
All immunotherapies used from disease onset, timing, and dosages	Two days post disease onset, IVIG (1g / kg/day for 3 days), 7 days post disease onset, glucocorticoid (IVMP 25 mg/kg/day for 3 days, 12.5 mg/kg/day for 3 days, and then continued with oral prednisone, which was then tapered down). Thirty-two days post disease onset, IVIG was repeated (2g / kg in total), and the first dose of tocilizumab (12 mg/kg) was given. Fifty-eight days post disease onset (tocilizumab 12 mg /kg), 85 days post disease onset (tocilizumab 12 mg/kg), 111 days post disease onset (tocilizumab 12 mg/kg), and 156 days post disease onset (tocilizumab 12 mg/kg).	Four days post disease onset IVIG (2g / kg in total), 6 days post disease onset glucocorticoid (IVMP 12 mg/kg/day for 3 days continued with oral prednisone and then tapered down), 18 days post disease onset (tocilizumab 8 mg/kg), 53 days post disease onset (tocilizumab 8 mg/kg), 87 days post disease onset (tocilizumab 8 mg/kg).	One day post disease onset IVIG (2g/kg in total), plus tocilizumab (12mg/kg), 2 days post disease onset glucocorticoid (IVMP 20mg/kg/day for 3 days then continued with oral prednisone and then was tapered down)	One day post disease onset (IVIG 2g/kg in total and was repeated at 57 days post disease onset), 11 days post disease onset, glucocorticoid (IVMP 4 mg/kg/day for 4 days continued with oral prednisone and then was tapered down), 35 days post disease onset (tocilizumab 12 mg/kg). Sixty-one days post disease onset (tocilizumab 12 mg/kg). Ninety days post disease onset (tocilizumab 10 mg/kg). 119 days post disease onset (tocilizumab 10 mg/kg), 147 days post disease onset (tocilizumab 10 mg/kg).
Disease duration before the first dose of IVIG	2 days	4 days	1 day	1 day
Disease duration before the first dose of IVMP	7 days	6 days	2 days	11 days
Disease duration before the first dose of tocilizumab	32 days	18 days	1 day	35 days
Effective therapy (es)	IVIG and IVMP (partially effective), tocilizumab (transient seizure control initially)	IVIG and IVMP (partially effective), tocilizumab (effective)	IVIG, IVMP, and tocilizumab (effective)	IVIG and IVMP (partially effective), tocilizumab (transient seizure control initially)
Number of tocilizumab cycles in the first half year of follow-up	5	3	1	5
Disease status before tocilizumab	Very frequent, 15 subclinical focal seizures recorded during the 2-hour EEG period	Frequent; dozens of seizures per day	The first seizure lasted for 90 minutes. However, he could have seizures seven times within 6 hours.	Not very frequent, 2-3 times a week
Possible tocilizumab side effects	None	None	Respiratory infection once.	None
Disease status half a year after the first dose of tocilizumab	No clinical or subclinical seizures	No clinical or subclinical seizures	No clinical or subclinical seizures	Rare short focal seizure (once to twice per month).
EEG status when in remission initially after tocilizumab treatment	Background: normal; no interictal epileptiform discharges and seizures	Background: slow with 4-5Hz theta rhythm; interictal epileptiform discharges: moderate diffused sharp slow wave, more localized in frontal and temporal lobes; no seizures	Background: slightly slow with 6-7Hz theta rhythm; no interictal epileptiform discharges and seizures	Background: normal; interictal epileptiform discharges: few episodic diffused slow waves; no seizures.
Disease situation at the last follow-up	Normal cognition but remained with chronic epilepsy (>50% reduction of seizure frequency)	Normal cognition and seizure freedom	Normal cognition and seizure freedom	Normal cognition but remained with chronic epilepsy (>50% reduction of seizure frequency).
Duration of follow-up	1 year and 5 months	1 year 2 months	1 year and 2 months	1year and 2 months

**Abbreviations:** EEG: electroencephalograph, LEV: levetiracetam, TPM: topiramate, VPA: sodium valproate, intravenous methylprednisolone, IVIG: intravenous immune globulin.

## Data Availability

All data generated or analyzed during this study are included in this published article.

## LIST OF ABBREVIATIONS

ASMs: Anti-Seizure Medications
CSF: Cerebrospinal Fluid
EEG: Electroencephalograph
FDA: Food and Drug Administration
NMOSD: Neuromyelitis Optica Spectrum Disorder
NORSE: New-Onset Refractory Status Epilepticus
TNF-α: Tumor Necrosis Factor Alpha

## STANDARDS OF REPORTING

STROBE guidelines were followed.
