# Supplementary material for: Blockade of Interleukin-6 Receptor with Tocilizumab During the Acute Phase of Childhood Immune-Mediated Epilepsy can Lead to a Favorable Outcome - an Observational Study
Source: Curr Neuropharmacol. 2026 Jan 15;24(6):867–86. doi: 10.2174/011570159X455475251203194508 (PMC13519841; doi:10.2174/011570159X455475251203194508)
Supplement: Supplementary file 1 [file CN-24-6-867_SD1.pdf]

Supplementary Material

Blockade of Interleukin-6 Receptor with Tocilizumab During the Acute Phase of Childhood Immune-Mediated Epilepsy can Lead to a Favorable Outcome - an Observational Study

Xiaolu Deng[1,2,3], Jielu Tan[1,2,3], Miriam Kessi[1], Zhanwei Zhang[1,2,3], Fang He[1,2,3], Ciliu Zhang[1,2,3], Fei Yin[1,2,3], Lifen Yang[1,2,3,*] and Jing Peng[1,2,3].

[1]*Department of Pediatrics, Xiangya Hospital, Central South University, Changsha, China;* [2]*Hunan Intellectual and Developmental Disabilities Research Center, Pediatrics, Changsha, China;* [3]*Clinical Research Center for Children Neurodevelopmental Disabilities of Hunan Province, Xiangya Hospital, Central South University, Changsha, China*

Table S1. Antibodies and complements levels in the serum of the patients.

	C4	C3	IgG	IgA	IgM
**Normal ranges**	100-400 mg/L	700-1400 mg/L	6.30-15.00g/L	460-2510 mg/L	210-530 mg/L
**Patient 1**	199	960	11.9	725	1150
**Patient 2**	70.2	846	22.2	373	1080
**Patient 4**	99.2	860	10.6	87.5	508

	IgA	Ig E	IgG	IgM	C3	C4
**Normal ranges**	0.82-4.53 g/L	0-165 I U/ml	7.51-15.6 g/L	0.46-3.04 g/L	0.79-1.52 g/L	0.16-0.38 g//L
**Patient 3**	0.98	< 5.00	5.84	2.52	0.97	0.27

1875-6190/26 $65.00+.00 © 2026 Bentham Science Publishers
